# Supplementary material for: HIVprotI: an integrated web based platform for prediction and design of HIV proteins inhibitors
Source: J Cheminform. 2018 Mar 9;10:12. doi: 10.1186/s13321-018-0266-y (PMC5845081; doi:10.1186/s13321-018-0266-y)
Supplement: Supplementary file 2 — Additional file 2. Source code of HIVProtI web server. [file 13321_2018_266_MOESM2_ESM.zip › HIVprotI_Source-code/hivprotI/weka-3-6-6/doc/weka/core/stemmers/NullStemmer.html]

NullStemmer


---


|  |  |  |
| --- | --- | --- |
| **Overview** \| **Package** \| **Class** \| **Tree** \| **Deprecated** \| **Index** \| **Help** \| **Weka's home** | |  |
| **PREV CLASS**   **NEXT CLASS** | **FRAMES**    **NO FRAMES**     **All Classes** |  |
| SUMMARY: NESTED \| FIELD \| CONSTR \| METHOD | DETAIL: FIELD \| CONSTR \| METHOD |  |


---


## weka.core.stemmers Class NullStemmer

```
java.lang.Object
  weka.core.stemmers.NullStemmer
```

**All Implemented Interfaces:**: java.io.Serializable, RevisionHandler, Stemmer

---

``` public class NullStemmer extends java.lang.Object implements Stemmer ```

A dummy stemmer that performs no stemming at all.

**Version:**
:   $Revision: 1.5 $

**Author:**
:   FracPete (fracpete at waikato dot ac dot nz)

**See Also:**: Serialized Form

---

**Constructor Summary**

`NullStemmer()`


| **Method Summary** | |
| --- | --- |
| `java.lang.String` | `getRevision()`             Returns the revision string. |
| `java.lang.String` | `globalInfo()`             Returns a string describing the stemmer |
| `static void` | `main(java.lang.String[] args)`             Runs the stemmer with the given options |
| `java.lang.String` | `stem(java.lang.String word)`             Returns the word as it is. |
| `java.lang.String` | `toString()`             returns a string representation of the stemmer |

**Methods inherited from class java.lang.Object**

`equals, getClass, hashCode, notify, notifyAll, wait, wait, wait`

**Constructor Detail**

### NullStemmer

```
public NullStemmer()
```


**Method Detail**

### globalInfo

```
public java.lang.String globalInfo()
```

:   Returns a string describing the stemmer

    :   **Returns:**: a description suitable for displaying in the explorer/experimenter gui

---


### stem

```
public java.lang.String stem(java.lang.String word)
```

:   Returns the word as it is.

    :   **Specified by:**: `stem` in interface `Stemmer`
    :   **Parameters:**: `word` - the unstemmed word **Returns:**: the unstemmed word, again

---


### toString

```
public java.lang.String toString()
```

:   returns a string representation of the stemmer

    :   **Overrides:**: `toString` in class `java.lang.Object`
    :   **Returns:**: a string representation of the stemmer

---


### getRevision

```
public java.lang.String getRevision()
```

:   Returns the revision string.

    :   **Specified by:**: `getRevision` in interface `RevisionHandler`
    :   **Returns:**: the revision

---


### main

```
public static void main(java.lang.String[] args)
```

:   Runs the stemmer with the given options

    :   **Parameters:**: `args` - the options


---


|  |  |  |
| --- | --- | --- |
| **Overview** \| **Package** \| **Class** \| **Tree** \| **Deprecated** \| **Index** \| **Help** \| **Weka's home** | |  |
| **PREV CLASS**   **NEXT CLASS** | **FRAMES**    **NO FRAMES**     **All Classes** |  |
| SUMMARY: NESTED \| FIELD \| CONSTR \| METHOD | DETAIL: FIELD \| CONSTR \| METHOD |  |


---
